# Anti-Inflammatory and Anti-Angiogenic Aattributes of *Moringa olifera* Lam. and its Nanoclay-Based Pectin-Sericin films

**DOI:** 10.3389/fphar.2022.890938

**Published:** 2022-08-25

**Authors:** Manal Ali Buabeid, Hafiza Sidra Yaseen, Muhammad Asif, Ghulam Murtaza, El-Shaimaa A. Arafa

**Affiliations:** ^1^ Department of Pharmacy, Fatima College of Health Sciences, Abu Dhabi, UAE; ^2^ Department of Pharmacy, COMSATS University Islamabad, Lahore Campus, Lahore, Pakistan; ^3^ Faculty of Pharmacy, Islamia University of Bahawalpur, Bahawalpur, Pakistan; ^4^ College of Pharmacy and Health Sciences, Ajman University, Ajman, UAE; ^5^ Department of Pharmacology and Toxicology, Faculty of Pharmacy, Beni-Suef University, Beni-Suef, Egypt; ^6^ Centre of Medical and Bio-Allied Health Sciences Research, Ajman University, Ajman, UAE

**Keywords:** tNF-alpha, inflammation, angiogenesis, Moringa olifera, interleukins

## Abstract

**Background:** Inflammation is a strong reaction of the non-specific natural immune system that helps to start protective responses against encroaching pathogens and develop typical immunity against intruding factors. However, prolonged inflammation may lead to chronic autoimmune diseases. For thousands of years, medicinal plants have served as an excellent source of treatment for chronic pathologies such as metabolic diseases.

**Purpose:** The present study aims to evaluate the anti-inflammatory and anti-angiogenic potential of *Moringa olifera* Lam. extract (*MO*) and *Moringa*-loaded nanoclay films.

**Methods:** The extract preparation was done through the maceration technique using absolute methanol (99.7%) and labelled as *Mo. Me*. *Mo. Me-*loaded nanoclay-based films were prepared by using pectin and sericin (Table 1). The *in vitro* studies characterized the film thickness, moisture, and phytochemical contents. The *in vivo* anti-inflammatory tests involved using a cotton pellet-induced granuloma model assay. In addition, the chick chorioallantoic membrane (CAM) assay was employed for angiogenesis activity.

**Results:** The phytochemical analysis of the extract confirmed the presence of alkaloids, glycosides, flavonoids and phytosterol. This extract contained quercetin in a large quantity. Cotton-pellet induced granuloma model study revealed a comparable (*p* > 0.05) effect of a high dose of *Mo. Me* (500 mg/kg) as compared with standard drug. Noteworthy, data obtained through the RT-PCR technique manifested the dose-dependent anti-oedematous effect of *Moringa olifera* via downregulation of TNF-α and interleukin-1ß. The findings of the CAM assay exhibited a remarkable anti-angiogenic activity of *Mo. Me* loaded nanoclay films, showing diffused vasculature network in the macroscopic snapshot.

**Conclusion:** *Moringa olifera* and its nanocomposite films have therapeutic potential against inflammation.

## 1 Introduction

Inflammation is a defensive response of a non-specific natural immune system, a set of complex response systems caused due to foreign pathogens, tissue injury, toxic chemicals, and trauma (Khalid et al., 2020), and a self-limiting healing process characterized by swelling of tissue, redness and pain. When inflammation becomes prolonged, it enhances tissue inflammation and contributes to various chronic autoimmune pathologies like rheumatoid arthritis (RA). Moreover, chronic inflammation associated with bone joint destruction causes damage to the surface and extracellular matrix of articular cartilage, which results in bone erosion if untreated. It hampers the quality of life (Saleem, Iftikhar, et al., 2021).

Inflammation is of two primary forms, i.e., acute and chronic. Acute inflammation is characterized as rapid onset, shorter duration, and transfer of antibodies from circulating blood to the site of injury and occurs due to stimuli such as hypoxia, ischemia, foreign bodies, bacterial, viral, and fungal infections, or toxins. This body response is a protective effect that leads to guarding the body against offending pathogens and can be reversible upon successfully eliminating a foreign body (Jeong et al., 2013). Chronic inflammation can last from several days to months. During this inflammatory reaction, tissue injury and repair occur simultaneously but at a different rate (Brücher & Jamall, 2019). So, the fluids rich in proteins and pro-inflammatory mediators try to permeate blood vessels from plasma, ultimately affecting blood vessels (Murakami & Hirano, 2012).

Different inflammatory mediators such as cytokines and free oxygen species are imperative in numerous chronic conditions such as rheumatoid arthritis, atherosclerosis, metabolic disorders and neurodegenerative diseases (Asif et al., 2016). It is along these lines essential to discover new therapeutic products that focus on inflammatory factors and are mainly associated with the pathogenesis of illness. Bradykinin is a product of α-globulin breakdown in plasma. Similarly, the kinin pathway has kininogenin through kallikreins which enhance vascular permeability by stimulating PGI-2 and nitric oxide secretion (Shukla, Rawal, & Jain, 2018). Bradykinin is the most active mediator of nociception. Its acts under the effect of prostaglandins and plays a role in spasmogenic action on uterine, bronchial and intestinal smooth muscles (Saleem, Asif, et al., 2021).

Pro-inflammatory cytokines like IL-1 and TNF-α are the pro-angiogenic markers. This information suggests that inflammation may induce angiogenesis (Jiang et al., 2020). Angiogenesis helps inflammation by transferring several inflammatory cells to the injured sites, and inflamed endothelium may be responsible for growth factors or cytokine secretion involved in disease initiation and progression (Yaseen et al., 2020). A sudden increase in angiogenesis rate during chronic inflammation often leads to an unwanted outcome. Cancer is also considered an injury that never heals completely (Shukla et al., 2018). Association between carcinogenesis and inflammation indicates a partial linkage with angiogenesis. Treatment with NSAIDs decreases the risk of multiple tumor burdens and cancer. Thus the anti-cancerous effects of NSAIDs could be attributed to their anti-angiogenic role (Asif et al., 2020).

The available anti-inflammatory therapeutic protocols include conventional corticosteroids and non-steroidal anti-rheumatic drugs (NSAIDs) (Wongrakpanich, Wongrakpanich, Melhado, & Rangaswami, 2018). However, profound adverse effects associated with these agents often lead to nephrotoxicity, gastrointestinal tract irritation and peripheral vascular diseases related to cardiac failure. Due to persistent symptoms, adverse effects, the cost involved in the current treatment protocol and an exploration of new, effective, and cheap anti-inflammatory products, alternative therapies, i.e., natural products, have drawn more attention nowadays. Plant products are considered better than synthetic substances due to their low cost, easy availability, and environment-friendly (Nisar, Sultan, & Rubab, 2018). An engineered drug delivery system may modify the biopharmaceutical properties of the active pharmaceutical agents (APIs) to achieve an immediate or delayed-release scenario as per therapeutic requirements. Notably, the programmed delivery of oral drugs to deliver medication at the site of action is valuable in chronotherapy of multiple pathologies (Yazdanpanah et al., 2022). Many plant products such as mucilage, gums and exudates are used as excipients in pharmaceutical dosage forms (Ullah et al., 2020). The traditional approach for managing pain by using NSAIDs two to three times a day does not allow an appropriate drug release which may result in patient noncompliance and poor therapeutic outcome (Auriemma, Cerciello, & Aquino, 2017).

*Moringa olifera* (*MO*), commonly known as the Miracle Tree, is native to Asia and Africa and widely grown in tropical and subtropical climates (Prajapati et al., 2022). *MO* tree belongs to the family *Moringaceae* and its leaves, roots, pods, flowers, and seeds have been extensively used to treat malaria, typhoid, cardiovascular and gastrointestinal disorders since ancient times (George et al., 2021). *MO* has several pharmacological effects, including gastro-protectant, hypotensive, antidiabetic, hepatoprotective, and antimicrobial (Fisall et al., 2021).

Therefore, nanocomposites have extensive use in drug delivery due to their unique characteristics, e.g. size, shape and high surface-volume ratio. Moreover, nanocomposites have multiple applications in various phases of science, including healthcare, optics, chemical industries, sensors, diagnosis and imaging (Ahmed, Ahmad, Swami, & Ikram, 2016). In the biomedical field, the nanoparticles-based pharmaceutical system is more dominant among all nanocomposites due to their unique characteristics, such as good antibacterial and antifungal activities (Bhavyasree & Xavier, 2020). Furthermore, few studies have reported the promising role of nanocomposites in wound dressings and cosmetic products as an antiseptic agent through disruption of enzymatic activities (Omidi, Sedaghat, Tahvildari, Derakhshi, & Motiee, 2018).

Therefore, the present research features a new approach to developing drug delivery systems potentially valuable for acute and chronic inflammatory disorders (Heinrich et al., 2020). This study aimed to explore anti-inflammatory and anti-angiogenic activity of *MO* through *in vitro* and *in vivo* tests. A particular focus was on the functionality of polymers in formulation design which is linked to the therapeutic outcome of extract in the body.

## 2 Materials and Methods

### 2.1 Materials

Pectin, sericin, nanoclay, and glycerol were purchased from Sigma Aldrich^®^, United States, while nutrient agar was acquired from CM 003, Oxoid, England, through the local distributors. All chemicals used in this study were of analytical grade.

### 2.2 Collection of plant material

Benessere Health Company, Multan, Pakistan provided the dried leaves of *Moringa olifera*. Dr Altaf Hussain Dasti from the Department of Botany, Bahauddin Zakariya University Multan, Pakistan, authenticated the identity of plant material and its voucher specimen was submitted to the university herbarium (Voucher No. 78X/20) for further experimentations.

### 2.3 Extract preparation

The leaves were finely powdered and placed in a container. Methanol was poured on top until it covered the whole powder. Then container was capped with an airtight cover and kept for the next 3 days. The material was stirred periodically to ensure complete extraction. Then, the micelle was separated from marc through filtration and dried using a rotary evaporator to make it solvent-free (Singh, 2008). The extract was stored in an airtight container at −4 C and labelled as *Mo. Me*.

### 2.4 Qualitative phytochemical screening of *Mo.Me*

Different identification tests (e.g., foaming test, Mayer’s reagent test, Wagner’s test, Hager’s test, alkaline reagent test, Shinodha test, ferric chloride test, lead acetate test, for oils and fats, Libermann-Burchard’s test, Borntrager’s test, and Keller Killani test) were performed for screening of secondary metabolites (phytosterol, saponins, alkaloids, glycosides and phenols) in *Mo. Me*.

### 2.5 Quantitative phytochemical analysis through high-performance liquid chromatography

The use of high-performance liquid chromatography (Shimadzu, Japan) helped quantitatively analyse phytochemicals present in *Mo. Me*. This technique used two types of mobile phases. Mobile phase-I comprised water and acetic acid in a ratio of 94:6 with pH = 2.27. On the other hand, mobile phase-II (15% acetonitrile) was run from 0 to 15 min followed by the use of 45% acetonitrile for 15–30 min and then 100% acetonitrile for 30–45 min. The isolation of compounds was done with the help of the Shim-pack HPLC (CLC-ODS; C-18) column (25 cm × 4.6 mm) having a 5 µM diameter. Samples analysis through an ultraviolet detector at 280 nm wavelength produced a chromatogram by plotting voltage (x-axis) and time (y-axis) units, respectively. Peaks of samples were compared with standards (Saleem et al., 2021).

### 2.6 Anti-inflammatory activities (cotton pellet granuloma model)

Albino rats of both sexes were purchased from The Riphah University Lahore, Punjab, Pakistan and kept under standardized conditions (relative humidity 50%), and temperature (25 ± 2°C). The supply of standard pellet diet and water was assured, and a 12 h light/dark cycle was maintained for all animals. Before the commencement of the study, animals were acclimatized for 1 week. The research Ethics Committee of COMSATS University Islamabad (CUI) approved the experiments and protocols followed for study conductance. The approval no. was 884/CUI/PHM-2021. Albino rats were divided into five groups (n = 5) for evaluation of anti-inflammatory activities, as given here; Group 1: Negative control (received normal diet and water); Group 2: Disease control (received distilled water 10 mg/kg orally); Group 3: Treatment-1 (administered 250 mg/kg *Mo. Me* orally); Group 4: Treatment-2 (administered 500 mg/kg *Mo. Me* orally) and Group 5: Standard (received indomethacin 10 mg/kg orally). Each group consisted of 3 male and 2 female rats.

After shaving the fur, the animals were anaesthetized. Sterile pre-weighed cotton pellets of 30 mg were implanted in the axilla region of each rat through a single needle incision (Umar et al., 2014). *Me. Mo* (250 and 500 mg/kg) and positive control (indomethacin 10 mg/kg) were administered to the respective group of animals for ten consecutive days from the day of cotton pellet implantation. On the 11th day, the animals were anaesthetized again; the cotton pellets were removed surgically and made free from extraneous tissues. The pellets were incubated at 37°C for 24 h and dried at 60°C to constant weight. The increase in the weight of dried pellets was regarded as a measure of granuloma formation. Percent inhibition of granuloma tissue formation was calculated using the reported formula (Sulaiman et al., 2010).

#### 2.6.1 Measurement of TNF-α and IL-Iß levels through RT-PCR technique

The inflammatory biomarkers TNF-α and IL-Iß were identified through a well-established technique “Real-Time Polymerase Chain Reaction” using QuantStudio3 Kits. Reagents were prepared as per the manufacturer’s instructions provided on the kit’s outlet. Lastly, the microplate was read on an automatic 96-well thermal cycler (Applied Biosystems Thermo Scientific^®^) at 450 nm to obtain the results (Yaseen et al., 2020).

### 2.7 Preparation of *Moringa olifera*-loaded nanoclay films

**FIGURE 1:**
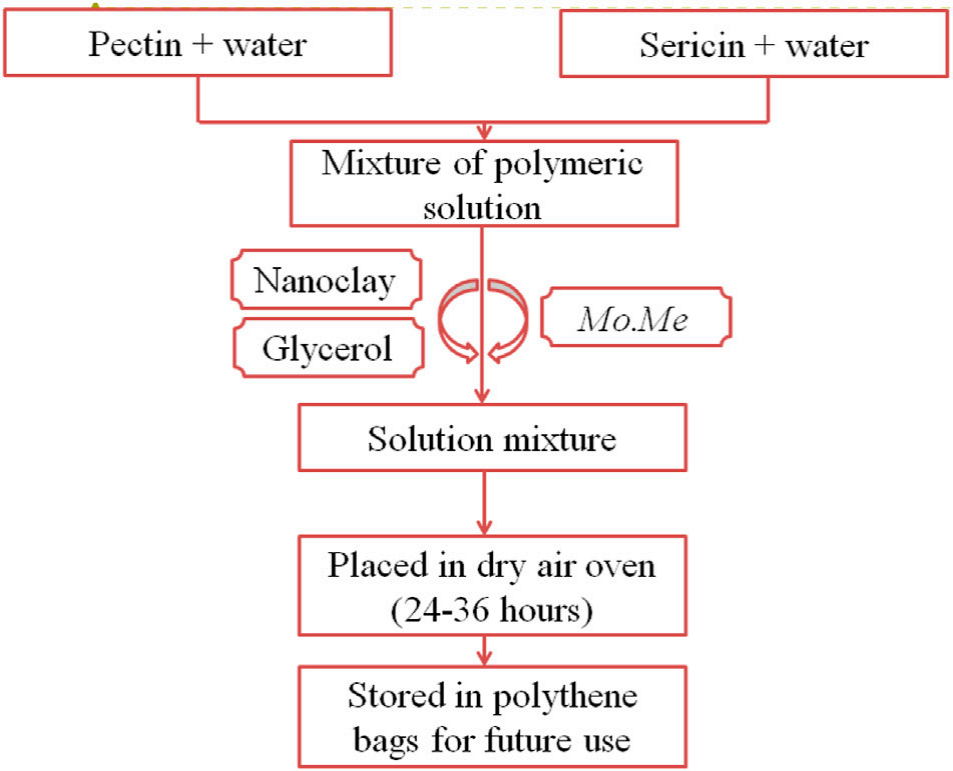
Preparation of *Moringa olifera* films.

*Mo.Me-*loaded nanocomposites were prepared by using pectin and sericin (Table 1). Glycerol was used to improve film flexibility. The collective heating and stirring technique prepared a mixture of the polymeric solutions (Shah, Yameen, Fatima, & Murtaza, 2019). Then, nanoclay and glycerol were added to the mixture (Figure 1). Moreover, the extract of *MO* was added to the solution mixture and dried in an oven at 37°C for 24–36 h. Films were separated from Petri-plates, wrapped in aluminium foil, and stored in polythene bags for further experimentation. The control films were similarly prepared in respective pectin-sericin solutions (Shah et al., 2019).

**TABLE 1:** Formulation table showing the pharmaceutical composition of control, nanoclay and *Mo.Me*-loaded nanoclay composites.

Disc name	Pectin (%)	Sericin (%)	Nanoclay concentration (Phr)	Moringa extract (Percentage of total polymer weight)	Glycerol (Percentage of polymer weight)
M1	100	0	3	20	40
M2	75	25	3	20	40
M3	50	50	3	20	40
M4	25	75	3	20	40
M5	50	50	3	40	40
M6	50	50	3	60	40
M7	50	50	3	-	-
M8	50	50	0	100	40

### 2.8 Physical analysis of films

#### 2.8.1 Film thickness

The average thickness of *Mo. Me* films were determined individually from various points of each formulation using a vernier calliper (Ullah et al., 2020).

#### 2.8.2 Moisture content

Moisture content (MC) of *Moringa* films was determined using a well-established method with slight modifications (Shah et al., 2019). Firstly, film pieces of uniform size were measured and placed in a dried air oven (105 C). After 24 h, the weight of film segments was measured again, and MC was obtained by using the following equation:

Percentage Inhibition=Wd-Wf/Wd×100

Where, Wd = initial weight of film segment, Wf = Final film weight.

### 2.9 Chicken chorioallantoic membrane assay

Forty fertile white leghorn chicken eggs were purchased from Batt hatchery, Sui-Asal road, Lahore and incubated for 3 days. On the 4th day, all eggs were cleaned with ethanol and incubated in a vertical position for 24 h at a temperature of 37°C and relative humidity 80%. Fertile eggs were classified into 11 groups so that groups 1–8 served as *Mo. Me* loaded nanoclay film treated group, group-9 and 10 served as *Mo. Me* (100 and 200 µg/ml dose) extract-treated group and 11th group was control (treated only with ethanol). Here, the 100 and 200 µg/ml of the extract were opted to explore the dose-dependent effect, not to standardize the dose. However, these doses were kept below 250 µg/ml, as discussed in the preliminary part of this study (results not presented here).

On the next day, a window was generated at the wide end of eggshells using sterile scissors and sterile discs of *MO* extract, *Mo. Me* loaded nanoclay films and ethanol-embedded Whatman’s filter paper discs were placed with the help of sterile forceps. The window was closed with paper tape and subjected to incubation for 48 h. After this, windows were reopened, and snaps were taken to analyse the results of the angiogenesis process in CAM of growing chick embryos (Yaseen et al., 2020).

### 2.10 Statistical analysis

Data were analysed using one-way ANOVA and post-hoc Tuckey’s test using Graph-Pad Prism Software. The results were presented as mean ± SEM (standard error of the mean) after statistical analysis through Graph-Pad Prism Software. The value of *p* below 0.05 was assumed to be significant.

## 3 Results

### 3.1 Physical analysis of films

Moisture contents indicate water in the composite film and have a primary role in maintaining the integrity, texture, quality, and mechanical characteristics of nano-composites. Water absorption increases with crystallinity. However, it is inversely proportional to modulus and tensile strength due to the plasticization effect (Rajeesh, Gnanamoorthy, & Velmurugan, 2010). Table 2 represents the highest crystallinity in M6 (50.4 ± 0.2) and M3 (50.1 ± 0.0) films followed by M8 (42.3 ± 0.2), M2 (41.52 ± 0.2), M1 (33.2 ± 0.1), M5 (33.6 ± 0.1), M4 (25.0 ± 0.1) and M7 (16.6 ± 0.0) in descending order. The formation of thin-film has become the subject of extensive research to understand the physical characteristics of drug-loaded films. As the volume of films increases, the distance between particles decreases which can reduce interchain entanglement (Bay et al., 2020). The thickness of M5 films was minimum, i.e. 1.02 ± 0.2 mm, while that of M4 nanocomposite films, thickness was maximum, i.e. 1.11 ± 0.2 mm (Table 2).

**TABLE 2:** Moisture content of *Mo.Me* nanoclay films.

Formulation code	Film thickness (mm)	Moisture content (%)
M1	1.03 ± 0.2	33.2 ± 0.1
M2	1.12 ± 0.1	41.5 ± 0.2
M3	1.04 ± 0.1	50.1 ± 0.0
M4	1.11 ± 0.2	25.0 ± 0.1
M5	1.02 ± 0.0	33.6 ± 0.1
M6	1.14 ± 0.1	50.4 ± 0.2
M7	1.11 ± 0.2	16.6 ± 0.0
M8	1.05 ± 0.2	42.3 ± 0.2

### 3.2 Qualitative phytochemical analysis

Table 3 shows primary and secondary metabolites present in *Mo. Me*. The amount of glycosides was meager. There was a slightly better amount of phytosterol and alkaloids in *Mo. Me*. However, *Mo. Me* contained an appreciable quantity of flavonoids and saponins.

**TABLE 3:** Phytochemical analysis of *Moringa olifera* methanolic extract.

Sr No.	Phytochemicals	Test name	Observations	Inferences
1	Alkaloids	Mayer’s test	Appearance of yellow precipitates	+
		Wagner’s test	Reddish-brown color precipitates	+
		Hager’s test	Yellow ppt	++
2	Saponins	Foam formation	Foam formation on vigorous shaking with distilled water	++
3	Fixed oils and fats	Solubility test	Presence of oily layer on aqueous layer	++
3	Glycosides	Borntrager’s test	Appearance of reddish-brown color	++
		Legal’s test		+
		Keller Killani test	Formation of greenish-blue color	−
4	Phyto-sterols	Liebermann-Burchard’s test	Grey color solution	+
5	Flavonoids	Alkaline reagent test	Yellow to colorless solution	+
		Shinoda test	Red color solution	−
		Ferric chloride test	Intense yellow color solution	+
		Lead acetate test	Appearance of yellow color	++

++ indicates heavy concentration, + slight concentration, − absent.

### 3.3 High-performance liquid chromatography

Quantitative phytochemical analysis revealed the presence of quercetin (3.15), gallic acid (4.48), caffeic acid (12.71), vanillic acid (13.33), benzoic acid (14.70) and p-coumaric acid (17.51). Moreover, the relative retention time of identified compounds is also described in Table 4.

**TABLE 4:** Compounds present in *Mo.Me* extract identified through HPLC.

Compound Name	Retention Time (min.)	Concentration (ppm)
Quercetin	3.15	13.30
Gallic acid	4.48	1.25
Caffeic acid	12.71	2.91
Vanillic acid	13.33	7.75
Benzoic acid	14.70	4.59
*p*-Coumaric acid	17.51	3.53

### 3.4 Anti-inflammatory activity (cotton pellet-induced granuloma assay)

**FIGURE 2:**
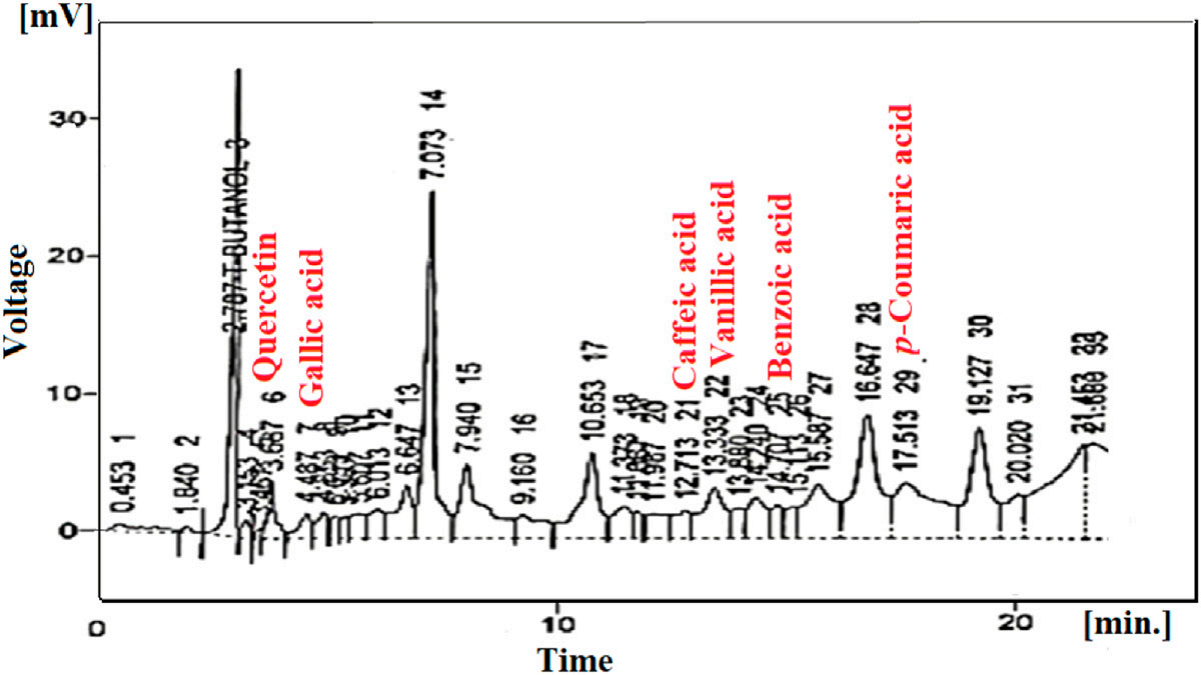
HPLC chromatogram of *Mo. Me* nanoclay films.

**FIGURE 3:**
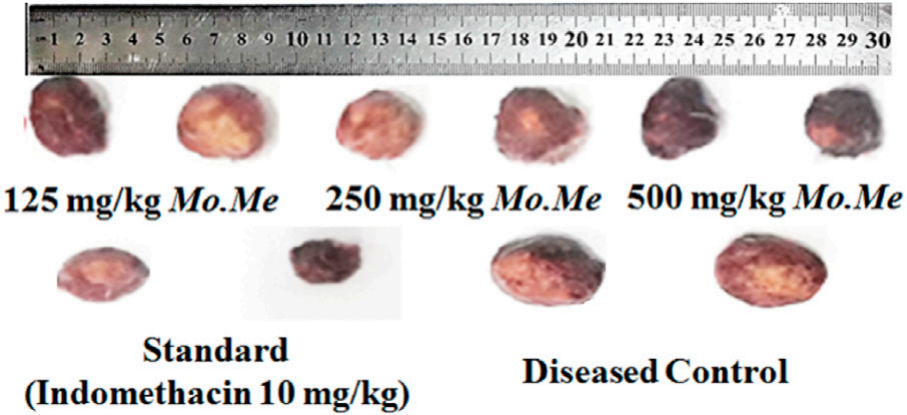
Macroanalysis of cotton pellets after treatment.

According to data of the cotton pellet model, percentage inhibition of granuloma formation in groups treated with *Mo. Me* (125, 250 and 500 mg/kg) and indomethacin (10 mg/kg) was 59.2 ± 5.45, 66.9 ± 4.21**,** 72.2 ± 2.51 and 76.5 ± 2.55, respectively. The comparison of *Mo. Me* 125 mg/kg treated groups showed a non-significantly (*p* > 0.05) low anti-inflammatory response, with the group receiving a dose of 250 mg/kg *Mo. Me*. While comparison of 250 mg/kg *Mo. Me* with 500 mg/kg also showed a non-significantly (*p* > 0.05) low effect against granuloma formation. Animals of the group treated with standard (10 mg/kg indomethacin), and the group receiving a high dose of *Mo. Me* (500 mg/kg) showed comparable (*p* > 0.05) inhibition of granuloma formation. Thus, different doses of *Mo. Me* showed dose-dependent anti-inflammatory activity with standard drug (Table 5; Figures 2A, 3).

**TABLE 5:** Anti-oedemtous effects of *Mo.Me* in cotton pellet induced granuloma model.

Group name	Inhibition (%)
Control (D.W)	0.0 ± 0.0
*Mo.Me* (125 mg/kg)	59.2 ± 5.4
*Mo.Me* (250 mg/kg)	66.9 ± 4.2ns
*Mo.Me* (500 mg/kg)	72.2 ± 2.5 ns
Standard (Indomethacin 10 mg/kg)	76.5 ± 2.5 ns

Values shown are mean ± SEM, of *Mo.Me* in cotton pellet induced granuloma study (*n* = 4). Where, ns = *p* > 0.05.

#### 3.4.1 Measurement of TNF-α and IL-Iß levels

According to Table 6, standard and *Mo. Me* treatment groups showed significant (*p* < 0.05) reduction in serum levels of TNF-α as 32.6 ± 0.5 (standard), 33.6 ± 0.4 (500 mg/kg), 34.5 ± 0.1 (250 mg/kg), 49.4 ± 0.6 (125 mg/kg) as compared to diseased control (66.2 ± 0.2). While treatment with *Moringa olifera* methanolic extract also downregulated serum concentration of IL-1ß, in following manner: 20.2 ± 1.0 (indomethacin), 23.5 ± 1.2 (500 mg/kg), 35.8 ± 0.8 (250 mg/kg), 44.3 ± 1.1 (125 mg/kg) and 45.5 ± 0.9 (control). Findings exhibited that indomethacin treatment group showed comparable (*p* < 0.001) effects with the highest dose of *Mo. Me* (500 mg/kg). Overall, RT-PCR results confirmed the dose-dependent anti-inflammatory effects of *MO* by decreasing concentration of inflammatory biomarkers as compared to diseased control (Table 6).

**TABLE 6:** Serum TNF-α and IL-1ß levels in different treatment groups.

Inflammatory Biomarkers	Diseased control	*Mo.Me* (125 mg/kg)	*Mo.Me* (250 mg/kg)	*Mo.Me* (500 mg/kg)	Standard (Indomethacin 10 mg/kg)
TNF-α (pg/ml)	66.2 ± 0.2	49.4 ± 0.6	34.5 ± 0.1	33.6 ± 0.4	32.6 ± 0.5
IL-1ß (pg/ml)	45.5 ± 0.9	44.3 ± 1.1	35.8 ± 0.8	23.5 ± 1.2	20.2 ± 1.0

### 3.5 Antiangiogenic assay

**FIGURE 4:**
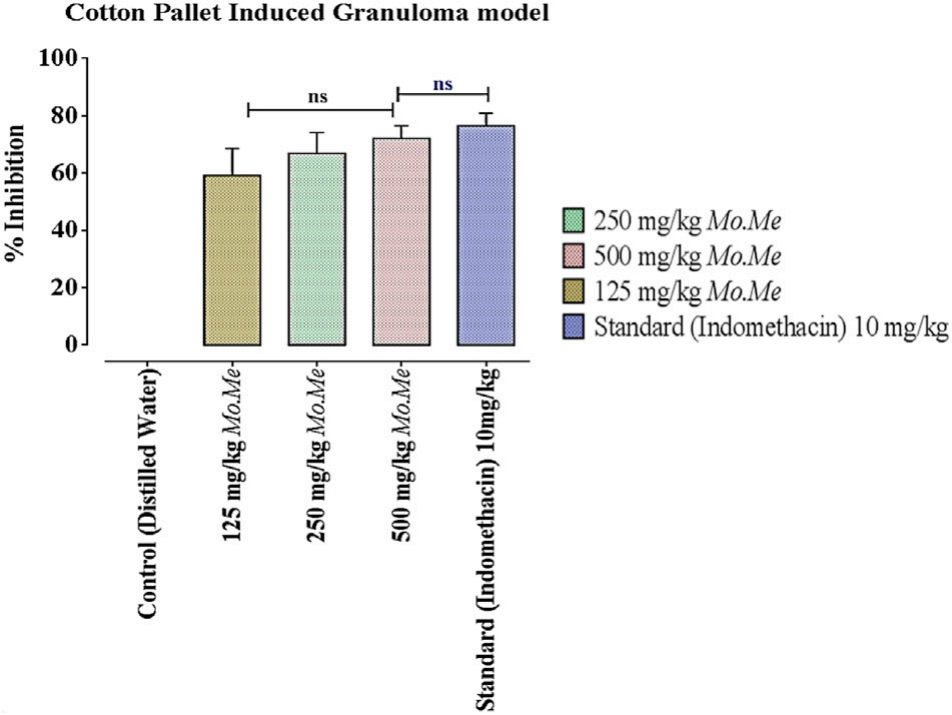
Effects of *Mo. Me* and standard drug on cotton pellet induced granuloma model. Values shown are Mean ± SEM of percentage inhibition of granuloma formation (*n* = 4). Where, ns = *p* > 0.05.

**FIGURE 5:**
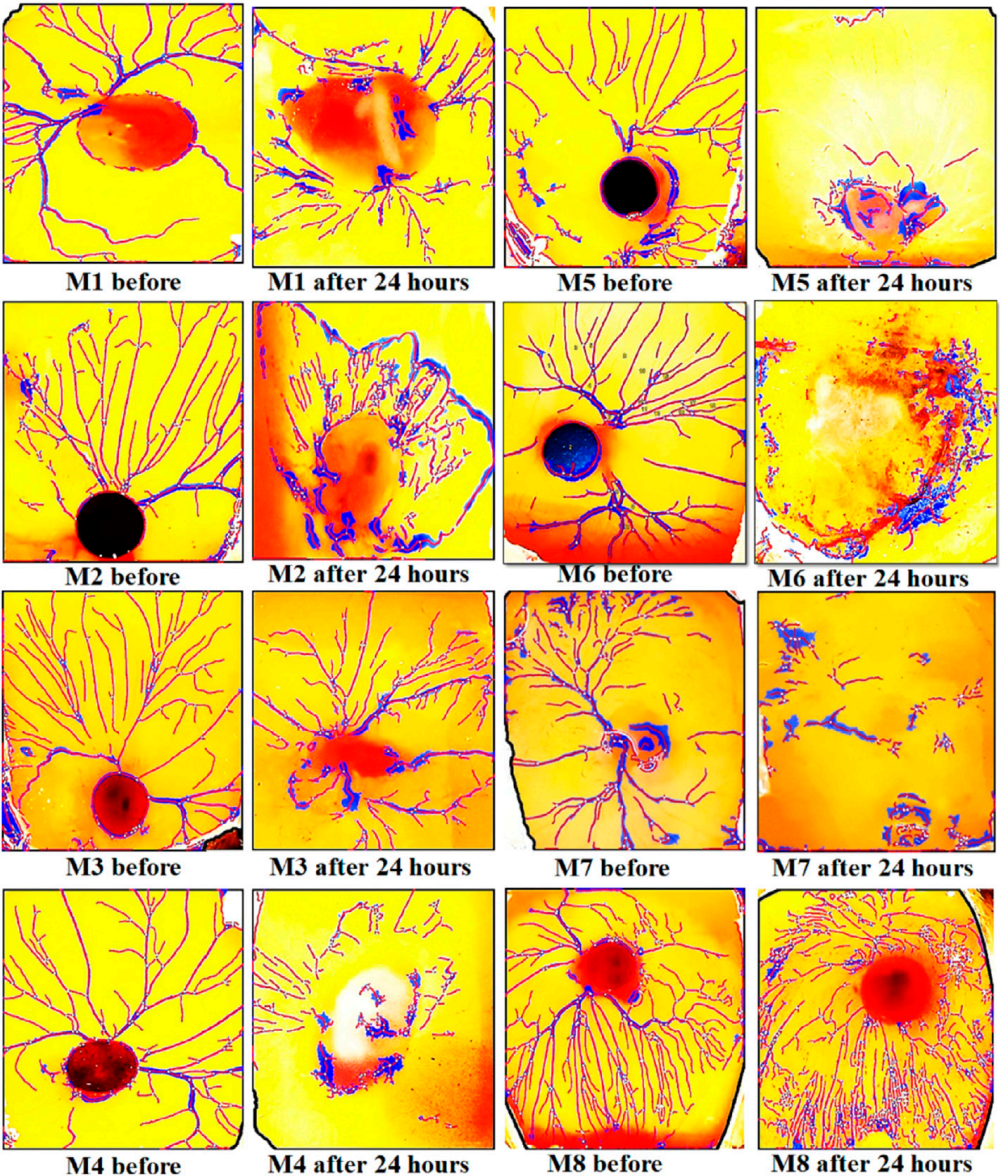
Results of CAM assay of *Moringa olifera* discs (1–8). The findings of the CAM assay showed a dissolved network of vessels in all types of *Mo. Me* loaded nano-composites.

**FIGURE 6:**
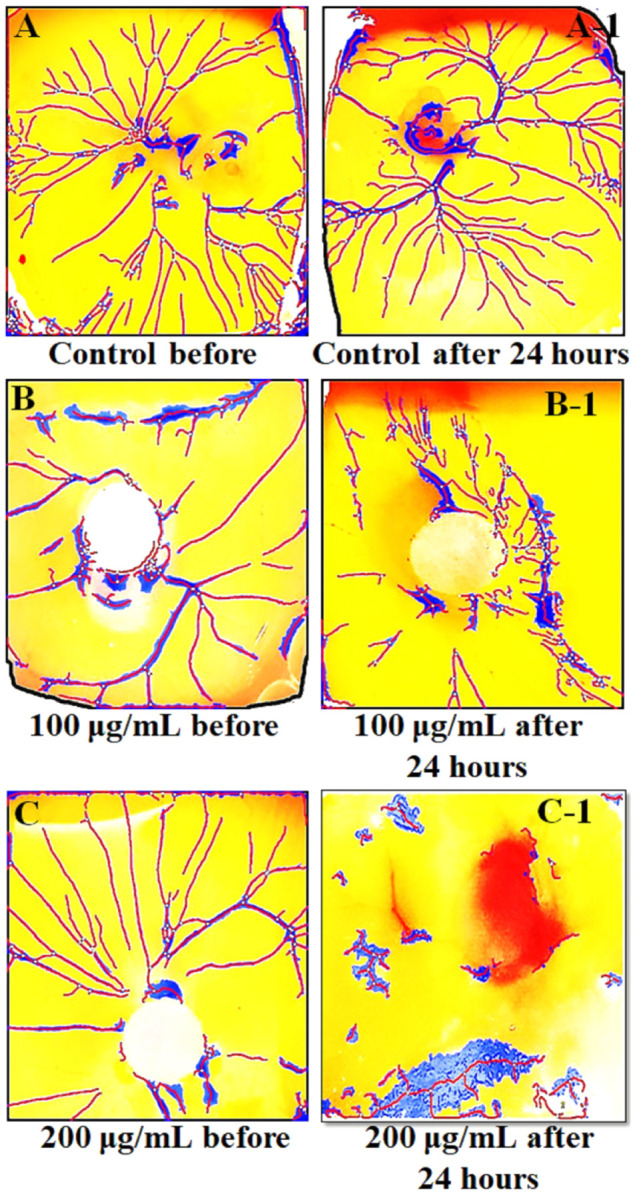
The results of CAM assay on two different concentrations (100 µg/ml and 200 µg/ml) of *Mo.Me*. **(A–C)** show the environment of eggs before treatment while A-1, B-1 and C-1 show the environment of eggs after treatment. Findings of CAM assay showed dispersed blood vessels network vessels upon treatment with methanolic extract of *Moringa olifera*.

Findings of *in vitro* CAM assay revealed that all *Mo. Me* loaded nanoclay films were having an anti-angiogenic effect. According to Table 7, M5 showed a significant (*p* < 0.001) anti-angiogenic effect, followed by M4, M7, M1, M3, M2, M6, and M8. The pure extract of *Mo. Me,* at a dose level of 200 µg/ml showed a profound (*p* < 0.01) effect on vascular network development and dissolved all blood vessels compared to the control group. While comparison of 200 µg/ml with 100 µg/ml showed comparable (*p* < 0.001) antiangiogenic effects after a period of 24 h (Figures 4–6; Table 7).

**TABLE 7:** Anti-angiogenic effect of *Mo.Me* nanoclay films on blood vasculature network.

Code	Blood vessels density [%]		Total blood vessels network Length [px]		Total branching points		Total nets		Total segments		Mean segment length [px]	
	0 h	24 h	0 h	24 h	0 h	24 h	0 h	24 h	0 h	24 h	0 h	24 h
M1	12.9	12.7	2,820	2,664	94	51	21	5	223	119	22	13
M2	18.9	14.0	3,883	3,428	116	98	16	6	271	215	18	13
M3	15.1	13.2	4,462	3,071	97	74	18	11	233	180	19	17
M4	12.6	8.3	3,447	2001	91	87	22	5	214	198	17	9
M5	13.9	6.8	3,809	1,253	101	34	20	8	254	90	14	15
M6	16.6	7.9	3,005	2,342	93	94	37	4	209	207	15	11
M7	15.2	9.2	3,428	1,789	95	59	20	19	246	149	14	12
M8	25.3	19.2	7,521	5,769	428	169	35	11	924	368	16	8
Control	16.3	16.6	4,149	4,502	115	148	4	8	231	302	15	18
*Mo.Me* 100 µg/ml	13.5	10.3	2,358	2,435	95	49	14	11	216	119	20	11
*Mo.Me* 200 µg/ml	12.6	11.6	2,596	2,929	117	48	14	10	263	110	27	10

## 4 Discussion

The present research was conducted to validate the folklore use of *MO* in autoimmune diseases like inflammation. In the modern pharmaceutical world, researchers continuously focus on formulating nanofilms, a versatile platform for drug delivery systems (Sarwar et al., 2020). Therefore, *Mo. Me*-loaded nanoclay films have been synthesized in the present research to check their therapeutic potential for inflammation-associated angiogenesis. Nanoclay is a layer of salicylates added to the formulation to enhance polymeric solutions’ mechanical and thermal behaviour. In contrast, glycerol has a role in film thickness and acts as a humectant/sweetener (Souza et al., 2012). Thin films can act locally and systemically and are convenient due to their easy swallowing, fast dissolution, and self-administrating properties (Karki et al., 2016). Secondary metabolites are considered bioactive agents in plants; therefore, to specify the therapeutic potential of methanolic extract of *MO* both qualitative and quantitative tests have been performed in the current study. Preliminary tests confirmed that *Mo. Me* was a great source of secondary metabolites. It was revealed that it contained a considerable quantity of saponins, alkaloids, flavonoids, and phytosterol while a slight concentration of glycosides (Ayu et al., 2020). Alkaloids are nitrogenous compounds with different pharmacological effects such as anti-inflammatory, antimicrobial, antidepressant, antihypertensive, antiviral, anti-emetic, diuretic, antitumor, antispasmodic, relaxant and sedative. Anti-inflammatory effects of alkaloids are produced by inhibition of COX, IL, TNF-alpha and NO (Wen et al., 2018; Peng et al., 2019). Flavonoids consist of naturally occurring polyphenolic compounds such as quercetin. Their effects as an antioxidant and anti-inflammatory agents are exhibited *via* inhibition of tumor necrosis-alpha, cyclooxygenases, reactive oxygen species, and interleukins. These are also involved in strengthening capillary walls and lowering the risk of cancer (Visweswari, Christopher, & Rajendra, 2013; Martín-Peláez et al., 2016). Glycosides are reported as muscle relaxants, sedatives, and diuretics (Visweswari et al., 2013). They inhibit the production of IL-1β, nitric oxide, and TNF-α, so they may have the potential to act as an anti-inflammatory and anticancer agent (Pongrakhananon, 2013; Yong et al., 2013). Saponins have anti-inflammatory, antiviral, antimicrobial, hypoglycemic, and anti-heptatonic effects (Visweswari et al., 2013) and inhibit TNF-alpha and COX-2 (Hassan et al., 2012; Jang et al., 2013). Phenols exert anticancer, antiseptic, anti-inflammatory and antioxidant effects (Visweswari et al., 2013) by inhibiting lipoxygenase and cyclooxygenase enzymes. Resultantly, this action suppresses production of inflammatory mediators (Ambriz-Pérez, Leyva-López, Gutierrez-Grijalva, & Heredia, 2016; Brezani, Smejkal, Hosek, & Tomasova, 2018). In the present study, the presence of above-mentioned secondary metabolites manifested anti-inflammatory as well as anti-angiogenic potential of *Mo. Me*.

Chronic inflammation is directly linked with tumour-associated angiogenesis, which leads to various diseases, including cancer. Epidemiological data show that more than 25% of all cancers are linked to un-cured inflammation (Brücher & Jamall, 2019). The link of inflammation with cancer implies two pathways, designated as extrinsic (by the release of chemicals, inflammatory problems alleviate cancer development) and intrinsic (mutations in the genes produce inflammation which leads to cancer) (Brezani et al., 2018). Likewise, cancer-associated inflammation contributes to tumor immune evasion characterized by leukocyte infiltration, which varies in size, distribution, and composition. Monocytes are involved in tumor from circulating blood by cancerous cells differentiating into macrophages by chemotactic factors like PDGF, VEGF and hypoxia (Sulaiman et al., 2010). Moreover, macrophages and other cells like lymphocytes, eosinophils, neutrophils, natural killer cells, dendritic and mast cells are involved in the production of multiple cytotoxic mediators like TNFα, IL-1ß and interleukins (Jeong et al., 2013; Dahmani & Delisle, 2018) having an imperative role in gene transcription, cell survival and proliferation. Therefore it can be briefed that impeding inflammation can stop abnormal angiogenesis and turn aside cancer (Behera & Bhattacharya, 2016).

Cotton pellet-induced granuloma was used to assess transudative, exudative, and proliferative components of chronic inflammation (Umar et al., 2014; Yang et al., 2020). When 3 hours have passed after pellet induction, vascular permeability of the vascular network increases which causes the immediate spillage of fluid and proteins present around blood vessels. From the 3rd to the 6th day, pro-inflammatory cytokines (pre-determinants of granulation tissue formation) are released continuously. In addition, reactive oxygen species and lysosomal enzymes also secrete and contribute to inflammatory pathway *via the* synthesis of collagen, fibroblasts formation and their penetration into exudate which will ultimately develop vascularized mass. The present study demonstrated dose-dependent reduction of granuloma formation (in terms of wet and dry weight) with the treatment of methanolic extract of *MO*, which is attributed to its excellent anti-inflammatory effect in the transudative and proliferative phase of inflammation. In addition, lower levels of inflammatory cytokines (e.g., tumor necrosis factor-alpha and interleukins-1ß) authenticated superb curative action of *MO* towards inflammation and its complications.

*In vitro* CAM assay is a simple and cheap alternative to animal models for determining tumor metastasis and angiogenesis. This assay has identified the anti-angiogenic and pro-angiogenic activity of any natural product, extract, or synthetic compound (Naik, Brahma, & Dixit, 2018). Findings of the present study revealed a pronounced inhibition of vasculature outgrowth in treatment groups indicating the anti-angiogenic potential of *Mo. Me*. Hence, the synergistic effect of phytochemicals and antioxidants of *Mo. Me* suggests that it has an excellent anti-angiogenic and anti-inflammatory potential. Because of the anti-inflammatory activities of *Mo. Me*, *Mo. Me* can be used as an anticancer agent. Besides this, polymer-based nanofilms serve as biocompatible interfaces and are promising as alternative bioactive medical devices that inherently resist chronic pathologies and dramatically control the release profile and stability of drugs (Lu et al., 2016). Hence, due to the unique properties, *Mo. Me* nanofilms may significantly increase the drug’s solubility, reduce cytotoxicity and improve therapeutic efficacy.

## 5 Conclusion

The percentage inhibition of granuloma formation and reduction in serum levels of TNF-α and IL-1ß indicate that *Moringa olifera* extract has a potential anti-inflammatory effect in a dose-dependent manner. In addition, *Mo. Me* loaded nanoclay film is found to be a potential vehicle for the delivery of *Moringa olifera* extract. To conclude, a positive correlation exists between *MO* extract and *Mo. Me* loaded nanoclay films in terms of therapeutic effect against inflammation. Further studies could be designed to explore the role of *MO* and its nanofilms against various inflammatory disorders, such as fatty liver, cancer, skin disorders and wound healing in humans.

## Data Availability

The original contributions presented in the study are included in the article/supplementary material, further inquiries can be directed to the corresponding authors.
